# Roles of Interaction between CCN2 and Rab14 in Aggrecan Production by Chondrocytes

**DOI:** 10.3390/ijms21082769

**Published:** 2020-04-16

**Authors:** Mitsuhiro Hoshijima, Takako Hattori, Eriko Aoyama, Takashi Nishida, Satoshi Kubota, Hiroshi Kamioka, Masaharu Takigawa

**Affiliations:** 1 Department of Orthodontics, Okayama University Graduate School of Medicine, Dentistry and Pharmaceutical Sciences, Okayama 700-8525, Japan; 2 Advanced Research Center for Oral and Craniofacial Sciences, Okayama University Dental School/Graduate School of Medicine, Dentistry and Pharmaceutical Sciences, Okayama 700-8525, Japan; 3 Department of Biochemistry and Molecular Dentistry, Okayama University Graduate School of Medicine, Dentistry and Pharmaceutical Sciences, Okayama 700-8525, Japan

**Keywords:** cellular communication network factor 2, CCN2, CTGF, Rab14, yeast two-hybrid, chondrocyte, ER stress, aggrecan

## Abstract

To identify proteins that cooperate with cellular communication network factor 2 (CCN2), we carried out GAL4-based yeast two-hybrid screening using a cDNA library derived from the chondrocytic cell line HCS-2/8. Rab14 GTPase (Rab14) polypeptide was selected as a CCN2-interactive protein. The interaction between CCN2 and Rab14 in HCS-2/8 cells was confirmed using the in situ proximity ligation assay. We also found that CCN2 interacted with Rab14 through its IGFBP-like domain among the four domains in CCN2 protein. To detect the colocalization between CCN2 and Rab14 in the cells in detail, CCN2, wild-type Rab14 (Rab14WT), a constitutive active form (Rab14CA), and a dominant negative form (Rab14DN) of Rab14 were overexpressed in monkey kidney-tissue derived COS7 cells. Ectopically overexpressed Rab14 showed a diffuse cytosolic distribution in COS7 cells; however, when Rab14WT was overexpressed with CCN2, the Rab14WT distribution changed to dots that were evenly distributed within the cytosol, and both Rab14 and CCN2 showed clear colocalization. When Rab14CA was overexpressed with CCN2, Rab14CA and CCN2 also showed good localization as dots, but their distribution was more widespread within cytosol. The coexpression of Rab14DN and CCN2 also showed a dotted codistribution but was more concentrated in the perinuclear area. Quantitative reverse transcription polymerase chain reaction (qRT-PCR) analysis revealed that the reduction in *RAB14* or *CCN2* mRNA by their respective siRNA significantly enhanced the expression of ER stress markers, *BIP* and *CHOP* mRNA in HCS-2/8 chondrocytic cells, suggesting that ER and Golgi stress were induced by the inhibition of membrane vesicle transfer via the suppression of CCN2 or Rab14. Moreover, to study the effect of the interaction between CCN2 and its interactive protein Rab14 on proteoglycan synthesis, we overexpressed Rab14WT or Rab14CA or Rab14DN in HCS-2/8 cells and found that the overexpression of Rab14DN decreased the extracellular proteoglycan accumulation more than the overexpression of Rab14WT/CA did in the chondrocytic cells. These results suggest that intracellular CCN2 is associated with Rab14 on proteoglycan-containing vesicles during their transport from the Golgi apparatus to endosomes in chondrocytes and that this association may play a role in proteoglycan secretion by chondrocytes.

## 1. Introduction

Cellular communication network factor 2/CCN family 2 (CCN2)/connective tissue growth factor (CTGF) is expressed in various types of cells, such as vascular endothelial cells, chondrocytes, and osteoblasts [1,2,3,4,5,6]. CCN2 regulates the cell proliferation, adhesion, migration, and differentiation of various types of cells, including the above cells, and is strongly expressed in growth plate cartilage, especially in (pre)-hypertrophic chondrocytes, enhancing bone formation through cartilage development [1,3,6]. CCN2 has multiple cellular functions, such as chondrocyte proliferation, the stimulation of the cartilage-specific extracellular matrix (ECM) synthesis, as well as chondrocyte maturation [1]. The importance of CCN2 in bone formation has also been demonstrated in *Ccn2* null mutant mice, which die within minutes after birth due to respiratory failure resulting from immature bone formation [7]; this indicates the essential role of CCN2 in chondrocytes.

CCN2 protein is composed of four characteristic modules of IGFBP-like (insulin-like growth factor binding protein-like), VWC (von Willebrand factor type C), TSP-1 (thrombospondin type 1) repeats, and CT (C-terminal cystine knot), and each domain has multiple interactive proteins by which CCN2 modulates the activity of these binding partners [1]. We previously reported that CCN2 binds to extracellular proteins, such as fibronectin through the CT domain [8] and aggrecan through the N-terminal half [9], and CCN2 also binds to CCN3 and CCN2 itself and modulates the activity each other [10].

There have also been reports suggesting that CCN5 may perform regulatory activities by acting as a transcriptional factor [11], and those interactions may play important roles in cellular functions. To identify additional intracellular, extracellular, or cell surface targets for CCN2 that may be regulated in the functions of CCN2 in chondrocytes, we searched for CCN2-binding proteins using the yeast two-hybrid screening system from a cDNA library derived from HCS-2/8, a human chondrocytic cell line [12]. We identified Ras-related protein (Rab) 14 GTPase as a new interactive protein of CCN2, suggesting that CCN2 may also exert intracellular functions by interacting to intracellular proteins.

Rab proteins belong to the low-molecular-weight GTPase superfamily and are involved in intracellular membrane trafficking [13]. These proteins are inactive when bound to GDP, but when the molecular switches are ON, they bind to GTP and change to the active form. Activated Rab proteins recruit “effector proteins” to the vesicle membrane and promote membrane trafficking. Different Rab proteins are localized to specific intracellular membranes, where they function as regulators of distinct steps in membrane traffic pathways [14]. Rab14 in particular is involved in membrane trafficking between the Golgi complex and endosomes and regulates apical targeting in polarized epithelial cells [15]. KIF16B reportedly associates directly with the Rab14 on FGF receptor containing vesicles and transports them toward the plasma membrane [16]. However, while the expression and function of Rab family small GTPases have been clarified using various types of cells, there has been only one report on the expression and function of Rab proteins in chondrocytes, which showed that Rab23 regulates chondrocyte differentiation in embryonal carcinoma-derived chondrogenic cell line, ATDC5 cells [17]. There have been no reports on Rab14 in chondrocytes.

There seems to be some functional interplay between CCN2 and Rab14; however, the functional and physiological interaction between these two proteins has not been explored. In the present study, we show for the first time that CCN2 interacts to Rab14 through its IGFBP-like domain among the four domains in CCN2 and indicate that CCN2 and Rab14 are colocalized in the cells and regulate intracellular membrane trafficking. We also found that endoplasmic reticulum (ER) and Golgi stresses were increased by suppressing the expression of mRNA of CCN2 and Rab14, resulted in the failure of the trafficking of aggrecan-containing vesicles from the Golgi apparatus.

## 2. Results

### 2.1. The Gene Expression of Rab14 and Ccn2 in Mouse Tissues

**Figure 1:**
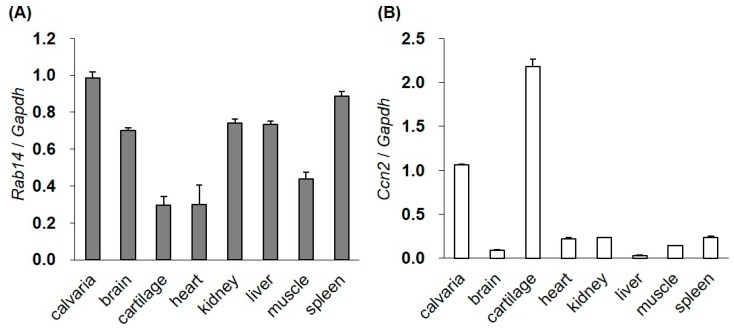
The gene expression of *Rab14* and *Ccn2* in mouse tissues. The expression of *Rab14* (**A**) and *Ccn2* (**B**) mRNA in the calvaria, brain, cartilage, heart, kidney, liver, muscle, and spleen tissues of four-day-old mice monitored by qRT-PCR. The level of each mRNA was standardized to that of *Gapdh* mRNA. The data are presented as the mean ± SD of triplicate samples.

We examined the expression of *Rab14* and *Ccn2* mRNA in various tissues from four-day-old mice by quantitative reverse transcription polymerase chain reaction (qRT-PCR). Although the expression of *Rab14* mRNA was high in the brain, kidney, liver, spleen, and calvaria bone, the mRNA was also observed in the rib cartilage, heart, and muscle (Figure 1A). *Ccn2* mRNA was strongly expressed in bone and cartilage (Figure 1B).

### 2.2. Rab14 Interacts with the IGFBP-like Domain of CCN2

**Figure 2:**
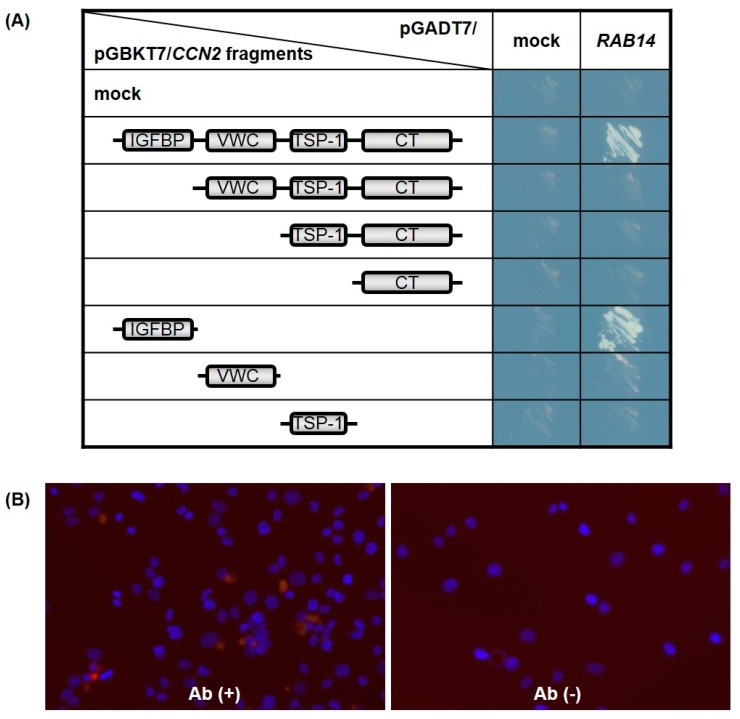
The cellular communication network factor 2 (CCN2) interaction domain with Rab14 GTPase (Rab14). Full-length CCN2 and its subdomains were expressed in the yeast cells as GAL4-BD fusion proteins, and full-length Rab14 was co-expressed as transcriptional activation domain fusion protein. The promoter activity induced by the interaction of both was monitored using independent reporter genes, such as histidine and adenine synthetase. Only transformants viable on synthetically defined medium lacking histidine and adenine were grown and visualized (mock: pGBKT7 or pGADT7 empty plasmid) (**A**). The interaction between CCN2 and Rab14 in HCS-2/8 cells was assessed by proximity ligation assay (PLA) technology (red, see result 2.2). Nuclei were visualized by DAPI (blue). *Abbreviations:* Ab (+), using α-CCN2 and α-RAB14 as primary antibodies; Ab (-), without primary antibody (negative control) (**B**).

Searching for CCN2 interactive proteins by yeast two-hybrid screening, we identified several clones from a human chondrocytic cell (HCS-2/8 cell) cDNA library that encoded Rab14, showing it to be an interactive protein of CCN2. To identify the interaction domains in CCN2, Rab14 was expressed in AH109 yeast cells as a GAL4-transactivation domain (GAL4-AD)-fusion protein, while the full-length and individual domains of CCN2 were coexpressed as GAL4-DNA binding domain (GAL4-BD)-fusion protein. The interaction between each CCN2 fragment and Rab14 led to the transcriptional activation of the GAL4 responsive promoter, and the promoter activity was monitored using independent reporter genes, such as histidine and adenine synthetase. As a result, only transformants viable on synthetically defined medium lacking histidine and adenine, respectively, were grown and visualized. Interaction with Rab14 was strongly observed after co-transformation with full-length CCN2 as well as a fragment containing the IGFBP-like domain (Figure 2A).

To confirm the interaction between CCN2 and Rab14 in chondrocytes, we next performed the in situ proximity ligation assay (PLA) using chondrocytic HCS-2/8 cells. In PLA, the target proteins of interest were recognized by two primary antibodies raised in different species. When the secondary antibodies called PLA probes were in close proximity, the protein interactions were visualized in fixed cells. As a result, we detected positive signals in the chondrocytic cells by using the combination of anti-CCN2 antibody (α-CCN2) and anti-RAB14 antibody (α-RAB14), and we hardly detected signals without these primary antibodies (Figure 2B). These results indicate that CCN2 and Rab14 interact in the chondrocyte, and the IGFBP-like domain contributed to the interaction between them.

### 2.3. Association of Intracellular CCN2 with Rab14 on Vesicles During Their Transport in Cells

**Figure 3:**
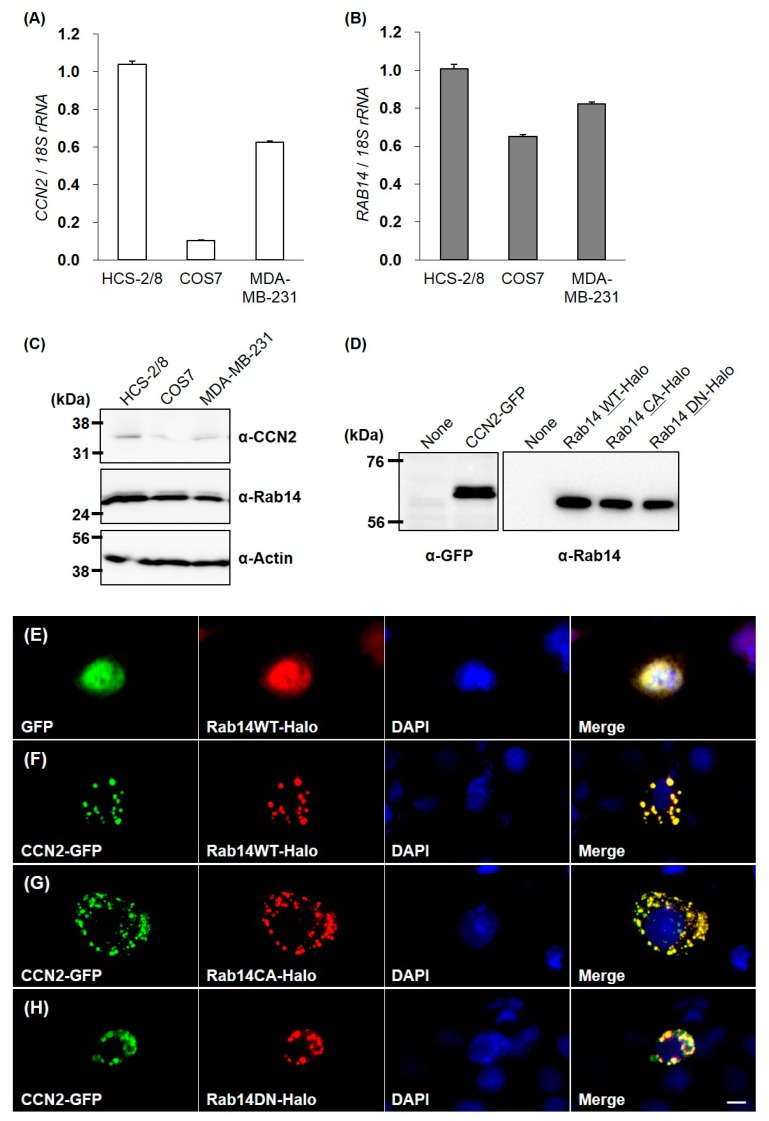
CCN2 and Rab14 expression in HCS-2/8, COS7, and MDA-MB-231 cell lines, and molecular imaging of CCN2 and Rab14 ectopically overexpressed in COS7 cells. The expression level of CCN2 and Rab14 in the cell lines was analyzed by qRT-PCR and Western Blotting (**A**–**C**). All cells were incubated under standard culture conditions (20% O_2_, 5% CO_2_, and 37 °C). The expression of GFP-fused CCN2, Halo-fused Rab14WT, Rab14 CA and Rab14DN proteins in the COS7 cell were confirmed by Western blotting (**D**). COS7 cells were cotransfected with GFP-fused CCN2 and Halo-fused Rab14WT or Rab14 mutants. GFP-fused proteins (green) and Halo-fused proteins (red) were overexpressed together and directly monitored by fluorescence microscopy. The counterstaining of nuclei was performed with DAPI (blue), and these images were merged using a software program. Without CCN2-coexpression, Halo-tag or Rab14WT showed cytosolic distribution (**E**). Halo-tagged Rab14WT was overexpressed with GFP-fused CCN2 (**F**). Rab14CA was overexpressed with CCN2 (**G**), and Rab14DN was overexpressed with CCN2 (**H**). Similar results were obtained in different examinations. Scale bar: 20 µm.

To confirm the interaction between CCN2 and Rab14 in living cells, we overexpressed green fluorescent protein (GFP)-fused CCN2 and Halo-fused Rab14 wild-type (Rab14WT) or Rab14 mutants ectopically. We first examined the expression of CCN2 and Rab14 in HCS-2/8, COS7 and MDA-MB-231 cells [18] by qRT-PCR and Western Blotting because we needed to know which cell line is suitable for this experiment. The basal levels of Rab14 expression did not vary remarkably among these cell lines. In contrast, the relative CCN2 expression in HCS-2/8 cells was 1.5- and 10-fold higher than MDA-MB-231 with a high ability of aggrecan production [18] and COS7 cells, respectively (Figure 3A–C). Preliminary experiment revealed that the strong positive expression of endogenous CCN2 masked the behavior of ectopic Rab14 in the cell. Therefore, to avoid such masking effect by endogenous CCN2, we selected COS7 cells that only slightly expressed and produced CCN2.

Next, we confirmed proper expressions of GFP-fused CCN2 and Halo-fused Rab14 proteins in the COS7 cells by Western Blotting (Figure 3D). Without GFP-fused CCN2 coexpression, Halo-fused Rab14 showed diffused cytosolic distribution (Figure 3E). However, when Halo-fused Rab14WT was overexpressed with GFP-fused CCN2, the Rab14 distribution changed to dots that were evenly distributed within the cytosol, and Rab14 and CCN2 showed complete colocalization (Figure 3F).

Previous studies showed that the overexpression of the constitutive active mutant Rab14Q70L promoted membrane trafficking between the Golgi complex and endosomes. Conversely, the overexpression of Rab14 dominant negative mutants Rab14S25N prevented such vesicle trafficking [15]. Therefore, we used these mutants to investigate the association between Rab14 and CCN2. When the constitutive active form Rab14Q70L (Rab14CA) was overexpressed with GFP-fused CCN2, Rab14 CA and CCN2 also showed good colocalization as dots, but their distribution was more widespread within the cytosol, as compared with those of wild-type Rab14 and CCN2 (Figure 3G). In contrast, the coexpression of the dominant negative form Halo-fused Rab14S25N (Rab14DN) and GFP-fused CCN2 also showed a dotted codistribution but was more concentrated in the perinuclear area (Figure 3H). These results suggest that intracellular CCN2 was associated with Rab14 on vesicles during their transport in cells.

### 2.4. Induction of ER and Golgi Stress by the Suppression of RAB14 or CCN2

**Figure 4:**
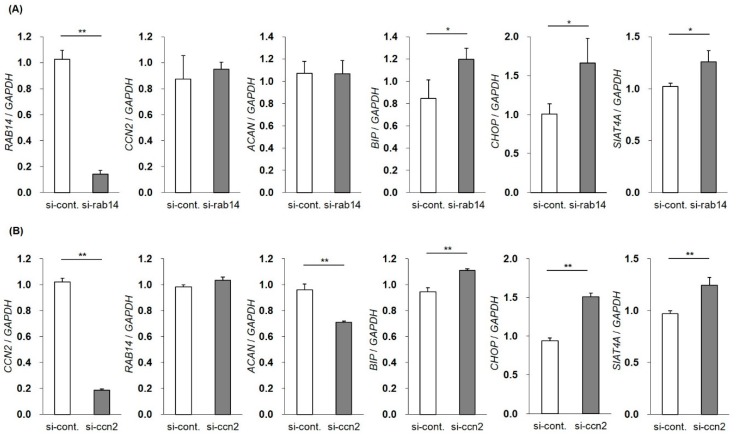
Changes in the gene expression by the silencing of the *RAB14* or *CCN2* gene. The expression of *RAB14* or *CCN2* mRNA was reduced using siRNA in HCS-2/8 cells, and the gene expression of *RAB14*, *CCN2*, *ACAN*, *BIP*, *CHOP* and *SIAT4A* mRNA was monitored by qRT-PCR. The mRNA expression profile of *CCN2*, *ACAN,* and stress marker genes in *RAB14* knockdown cells (**A**). The mRNA expression profile of *RAB14*, *ACAN,* and stress marker genes in *CCN2* knockdown cells (**B**). The level of each mRNA was standardized to that of *GAPDH* mRNA. The data are presented as the mean ± SD of triplicate samples (*t*-test * *P* < 0.05, ** *P* < 0.01).

To investigate the roles of intercellular CCN2 interacted with Rab14 in membrane trafficking in chondrocytes, we decreased the expression of *RAB14* mRNA using siRNA in HCS-2/8 cells and monitored the gene expression of *CCN2*, *ACAN*, *BIP*, *CHOP,* and *SIAT4A* by qRT-PCR. The suppression of *RAB14* mRNA enhanced the expression of *BIP*, *CHOP,* and *SIAT4A* mRNA, suggesting that the inhibition of membrane trafficking resulted in ER and Golgi stress. However, the inhibition of *RAB14* mRNA did not alter the gene expression of *CCN2* and *ACAN* mRNA (Figure 4A). Similarly, we reduced the gene expression of *CCN2* and monitored the *RAB14*, *ACAN*, *BIP*, *CHOP,* and *SIAT4A* mRNA expression. The expression of *BIP*, *CHOP,* and *SIAT4A* mRNA was significantly enhanced. The suppression of *CCN2* did not change the *RAB14* mRNA expression but decrease the *ACAN* mRNA expression (Figure 4B). These data suggest that ER and Golgi stress were induced by the inhibition of membrane–vesicle transfer via the suppression of CCN2 or Rab14.

### 2.5. Reduction in Aggrecan Accumulation in HCS-2/8 Cells by Rab14DN Overexpression

**Figure 5:**
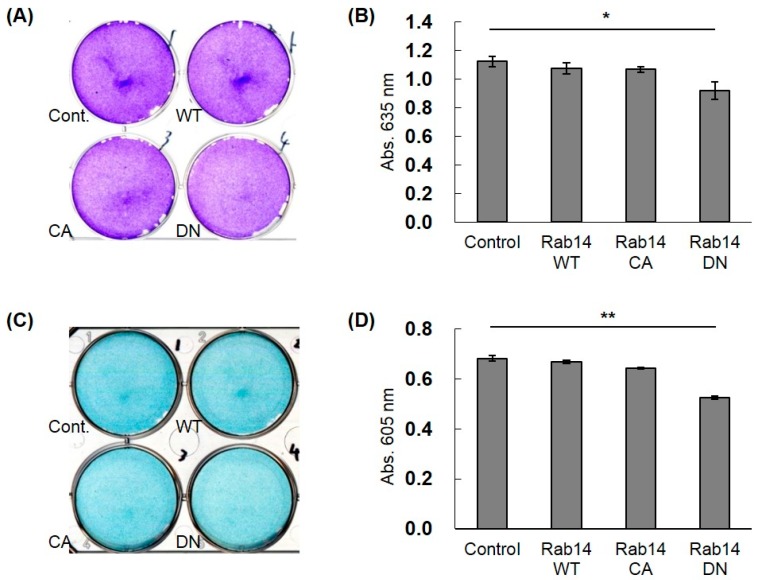
The effect of Rab14 on the accumulation of ECMs in HCS-2/8 cells. Halo (Cont.), Rab14WT, Rab14CA, and Rab14DN expression vectors and the control vector were transfected in the HCS-2/8 cells and cultured for 7 days, and then stained with toluidine blue (**A**) or alcian blue (**C**). The staining intensity was quantified by measuring the absorbance at 635 or 605 nm with a spectrophotometer (**B**,**D**). The data are presented as the mean ± SD of triplicate samples (*t*-test * *P* < 0.05, ** *P* < 0.01).

To study the effects of the interaction of CCN2 and Rab14 on the secretion of ECMs in chondrocytes, we transfected HCS-2/8 cells with Rab14WT, Rab14CA, Rab14DN expression vectors, and Halo expression vector (control) and monitored the accumulation of aggrecan, which is a major ECM in cartilage, by toluidine blue (Figure 5A) or alcian blue (Figure 5C) staining. Rab14DN overexpression in HCS-2/8 cells reduced the proteoglycan accumulation to less than 75% of that with Rab14WT overexpression in both staining protocols (Figure 5B,D). Furthermore, when the expression of *RAB14* mRNA was reduced using siRNA in HCS-2/8 cells, we monitored the accumulation of aggrecan. The suppression of *RAB14* mRNA decreased the accumulation to less than 87% of that with control siRNA treatment in Alcian Blue staining (data not shown).

## 3. Discussion

Rab GTPase is a master regulator that establishes secretory and endocytic pathways. The role of Rab proteins is to recruit binding proteins called effectors, such as adaptor factors and motor proteins, to facilitate downstream intracellular membrane trafficking [19]. Rab33B modulates autophagosome formation through interaction with Atg16L [20]. Rab27A binds to Slp1/JFC1 in neutrophils and interacts with Slp4/granuphilin in pancreatic β cells. These interactions are thought to promote the targeting of the granules to the plasma membrane [21,22]. More than 60 Rab isoforms have been reported in mammals, including humans and mice. There are several effector molecules for each Rab isoform, and these effectors exhibit broad Rab binding specificity, binding to multiple Rab isoforms [23,24]. For example, the direct interaction of Rab14 with PKCι reportedly modulates the epithelial barrier function through the regulation of claudin-2 [25], and its binding to KIF16B promotes the trafficking of the FGFR-containing vesicles to the plasma membrane [16].

In the present study, we determined the expression of *Rab14* in cartilage (Figure 1) and found that Rab14 was a new interactive protein of CCN2, which is abundant in cartilage (Figure 2 and Figure 3). CCN2 is a secretory protein that accumulates at the cell surface and binds various extracellular matrix components, such as fibronectin [8] and aggrecan [9]. CCN2 also interacts with several growth factors, including TGF-β and BMP-4 [26], and thereby modulates their activities. Among CCN family members, CCN3 directly interacts with CCN2 as a regulator of CCN2 in chondrocytes [10]. Although a few other CCN member proteins are found in nuclei [11,27], CCN proteins, including CCN2, are generally believed to manipulate extracellular signaling. In particular, there have been no reports showing that CCN2 functions inside the cells. In the present study, we demonstrated for the first time the interactions of Rab14 with CCN2 and provided evidence suggesting that the complex regulates intracellular membrane trafficking in chondrocytes.

Using its four modules, CCN2 regulates the molecular network by manipulating these molecular counterparts [28]. The specificity of interaction between Rab14 and CCN2 in the present study was substantiated by the yeast two-hybrid assay, which revealed that Rab14 interacts to the IGFBP-like domain of CCN2 (Figure 2A). Furthermore, the interaction between CCN2 and Rab14 in HCS-2/8 cells was confirmed using in situ PLA (Figure 2B). The participation of Rab14 in trafficking between the Golgi complex and early endosomes has been reported [15]; however, its specific role in membrane trafficking is currently unknown. When Rab14WT was overexpressed without GFP-fused CCN2, Halo-tagged Rab14 showed a diffuse cytosolic distribution in the cells (Figure 3E). When Rab14WT and CCN2 were co-overexpressed, both molecules were almost completely colocalized and distributed as dots within the cytosol (Figure 3F); these data indicate that CCN2 accelerates the dot-like association of Rab14. Moreover, when Rab14CA and CCN2 were co-expressed, more widespread distribution of the dots showing their co-localization was observed within the cytosol (Figure 3G). In contrast, when Rab14DN and CCN2 were co-expressed, more concentrated dots showing their co-localization were observed in the perinuclear area (Figure 3H). Taken together, these findings suggest that intracellular CCN2 enhances the accumulation of Rab14 on vesicles through the IGFBP-like domain of CCN2 during their transport within the cytosol, and CCN2 may cooperatively regulate endocytotic trafficking with Rab14 as an effector protein.

The interactions between Rab GTPases and their effector proteins are critical events in vesicular trafficking. Various lines of evidence suggest that defects in vesicular transport mechanisms lead to ER stress [29], probably induced by the stagnation of proteins in the ER due to incompetent protein trafficking. As shown in Figure 4, we observed that the silencing of *RAB14* or *CCN2* mRNA respectively promoted the expression of ER or Golgi stress-related genes, such as *BIP*, *CHOP,* and *SIAT4A*, in chondrocytic cells. The present findings suggest that a reduction in the activity of either Rab14 or CCN2 may cause ER or Golgi stress, accompanied by the failure of intracellular trafficking. Furthermore, the ER stress derived from the stagnation of proteins within the cytosol may alter the secretion of ECM in cartilage. Therefore, it is feasible that the overexpression of Rab14DN restricts vesicular trafficking (Figure 3H). These data suggest that the cytosolic distribution of matrix-containing vesicles was inhibited by Rab14DN. The stagnation of vesicular trafficking would therefore enhance the basal ER stress and influence the proteoglycan release and accumulation. We have reported that CCN2 stimulates the mRNA expression of aggrecan in chondrocytic cells [1,2,3] and siRNA against the *CCN2* down-regulated mRNA expression of aggrecan in chondrocytic cells [30,31]. However, Rab14 is a protein involved in intracellular membrane trafficking and not a signal inducer, so it is not surprising that Rab14 siRNA had no effect on *ACAN* mRNA expression. Considering the role of Rab14, it is feasible that Rab14 siRNA rather inhibits the intracellular transport of aggrecan, resulting in an accumulation of aggrecan in the cytosol. For this reason, Rab14DN overexpression in chondrocytes reduce the proteoglycan accumulation to a greater degree than Rab14WT overexpression (Figure 5). Concerning the results that the CCN2-silincing caused ER and Golgi stress (Figure 4B), it is noteworthy that primary chondrocytes from CCN2 deficient mice also showed reduced proteoglycan accumulation [32].

**Figure 6:**
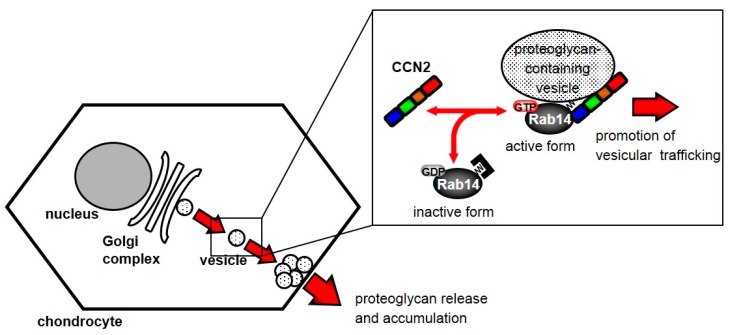
Schematic diagram showing the behavior of Rab14 and CCN2 in chondrocytes. Cytosolic Rab14 is recruited to the Golgi complex by the association with intracellular CCN2 and transports proteoglycan-containing vesicles from the Golgi complex to endosomes in chondrocytes.

Based on the present findings, we propose a model for the regulation of vesicle trafficking by the interaction between CCN2 and Rab14. Activated cytosolic Rab14 interacts with CCN2 as an effector protein, and Rab14 associates with proteoglycan-containing vesicles. Consequently, this interaction promotes vesicle trafficking. As a result, the Rab14-CCN2-associated vesicles promote the secretion of proteoglycan into the extracellular space (Figure 6). Suppressing this trafficking induces ER and Golgi stress through the failure of trafficking of proteoglycan-containing vesicles from the Golgi complex. These results suggest that the interaction between CCN2 and Rab14 plays a critical role in the chondrocytic function.

## 4. Materials and Methods

### 4.1. Cell Culture

HCS-2/8, a human chondrosarcoma-derived cell line, MDA-MB-231, a human-breast-cancer-derived cell line and COS7, a monkey kidney cells were cultured in Dulbecco’s Modified Eagle Medium (DMEM) supplemented with 10% fetal bovine serum (FBS), as described previously [12,18,33,34]. All cells were incubated under standard culture conditions (5% CO_2_ and 37 °C).

### 4.2. Total RNA Extraction, Reverse-Transcription, and qRT-PCR

Calvaria, brain, cartilage, heart, kidney, liver, muscle, and spleen tissues from four-day-old mice were homogenized, and these total RNAs were extracted using the RNeasy Mini Kit (Qiagen, Hilden, Germany). The total RNA of the cell lines was also extracted by the same way. These RNAs were reverse-transcribed with avian myeloblastosis virus reverse transcriptase (Takara Bio, Shiga, Japan) at 37 °C for 15 min and subjected to qRT-PCR (StepOnePlus; Applied Biosystems, Foster City, CA, USA) using SYBR Green Realtime PCR Master Mix (Toyobo, Osaka, Japan). All operations were performed according to each manufacturer’s instruction. The sequence of used primers is reported in Table 1 and Table 2. The mRNA expression was normalized to the expression of the housekeeping gene *Gapdh* or *18S rRNA*.

### 4.3. The Analysis of CCN2 Domain Interacting to Rab14

We performed yeast two-hybrid screening using a cDNA library derived from HCS-2/8 cells as described previously [9,35]. For the investigation of the CCN2 domain interacting with Rab14, the expression plasmid of full-length Rab14, which was expressed as a GAL4AD-fusion protein, and plasmids including CCN2 fragments, which were co-expressed as GAL4BD-fusion proteins, were used to transform AH109 yeast cells. The interaction was monitored by checking the growth of yeasts on -Ade/-His/-Leu/-Trp synthetically defined medium. The primers used for the amplification of the full-length and truncated forms of *CCN2* are shown in Table 3.

### 4.4. In Situ Proximity Ligation Assay (PLA)

The CCN2 and Rab14 interaction in HCS-2/8 cells was examined using Duolink™ In Situ PLA (Merck, Darmstadt, Germany). HCS-2/8 cells (200 μL of a suspension with 1 × 10^5^ cells/mL) in DMEM/10%FBS were prepared on cover glasses and incubated for 24 h at 37 °C. The cells were fixed with 4% PFA for 15 min. All remaining operations were performed according to manufacturer’s instruction and a previous report [36]. Anti CTGF Module 3, Monoclonal Antibody (3-54) (Wako, Osaka, Japan) and Anti-RAB14 antibody ab40938 (abcam, Cambridge, UK) were used as primary antibodies. The images were acquired using a fluorescence microscope (BZ-9000) (KEYENCE, Osaka, Japan).

### 4.5. Expression Vectors

We prepared pEGFP/*ccn2* vector, expressing CCN2 protein with GFP at the C-terminus, by amplification of *CCN2* cDNA with signal peptide using the primers 5′-cttcgaattcccatgaccgccagtatgggccccgtc-3′ and 5′-cggtggatcccgtgccatgtctccgtacatcttcctgta-3′ with insertion into the EcoRI and BamHI sites of the pEGFP-N1 vector. We also prepared pFlag-CMV/*RAB14*-*HALO* expressing Rab14WT protein with a Halo-tag at the C-terminus of Rab14WT that was prepared by the amplification of *RAB14* cDNA using the primers 5′-atccaagcttatggcaactgcaccatacaactactc-3′ and 5′-atacgaattcgcgcagccacagccttctctctg-3′ and by the amplification of *HALO* [37]. The *HALO* fragment was cloned into the pFlag-CMV vector at the EcoRI/BamHI sites, and the RAB14 fragment was cloned into the pFlag-CMV/*HALO* vector at the HindIII/EcoRI sites. Rab14CA (Q70L) and Rab14DN (S25N) mutants were generated using a PCR mutagenesis kit (TaKaRa Bio, Shiga, Japan) with the pFlag-CMV/*RAB14*-*HALO* as a template.

### 4.6. Western Blotting

The lysate of HCS-2/8, COS7, and MDA-MB-231 cells was analyzed by Western Blotting with an antibody against CCN2 (R&D Systems, Minneapolis, MN, USA), Rab14 (Santa Cruz, Dallas, TX, USA), and β-Actin (Sigma, St Louis, MO, USA), respectively. The COS7 cells that were transfected with pEGFP⁄*ccn2* or pFlag-CMV⁄*RAB14-HALO* (wt or mutants) were incubated for 48 h. The lysates were harvested and analyzed by Western Blotting with an antibody against GFP or an antibody against Rab14. Western Blot analysis was performed as described previously [5].

### 4.7. Fluorescence Imaging

We transiently transfected COS7 cells with different expression vectors and treated them with the fluorescent ligand 2,6-dideoxy-4-thiomethyl-β-D-ribohexopyranoside (TMR), which recognize HaloTag (Promega, Madison, US, USA), for 15 min at 37 °C, fixed them with 4% formaldehyde (PFA)/phosphate-buffered saline (PBS) for 15 min at room temperature (RT), and then mounted them on slides with Prolong Gold Mounting Medium containing 4′6-diamidino-2-phenylindole (DAPI) nuclear stain (Life Technologies, Waltham, MA, USA). GFP-tagged proteins, Halo-tagged proteins, and nuclei were directly monitored by fluorescence microscopy using an IX70 Microscope (Olympus Corporation, Tokyo, Japan). The images were analyzed with the Axiovision software program (Zeiss, Oberkochen, Germany).

### 4.8. Indirect Immunofluorescence and Fluorescence Deconvolution Microscopy

HCS-2/8 cells were fixed with 4% PFA/PBS for 15 min at RT, permeabilized with 0.25% TritonX-100/PBS or not permeabilized for 8 min and then blocked with 5% skim milk in PBS for 1 h followed by incubation with primary antibodies for 90 min at RT. After being incubated with secondary antibodies, images of the cells were obtained with an Olympus fluorescence microscope.

### 4.9. Small Interfering RNA (siRNA) Experiments

The expression of *RAB14* and *CCN2* was knocked down by RNA interference. MISSION^®^ siRNA directed against human Rab14 and CCN2 was purchased from Merck (Darmstadt, Germany). MISSION^®^ siRNA Universal Negative Control #1 was also purchased from Merck and used. HCS-2/8 cells were transfected with 30 nM of siRNA and seeded onto 6-well multiplates at 70–80% confluence. At 24 h after transfection, total cellular RNA was harvested and evaluated for the expression of each gene.

### 4.10. Toluidine Blue and Alcian Blue Staining

HCS-2/8 cells in suspension, which transiently transfected with different expression vectors or treated the siRNA, were seeded onto 6-well multiplates at 70–80% confluence and cultured for 7 days. The culture medium was then removed, and the cells were washed with PBS. These cells were fixed with paraformaldehyde (PFA) solution for 30 min at RT. The fixed cells were stained with 0.05% toluidine blue solution or 1% alcian blue solution for 20 or 60 min at room temperature and then washed three times with PBS. To quantify the staining intensity of the toluidine blue- and alcian blue-stained matrix, the stained aggrecan was extracted with lysis buffer. The optical density of the extracted dye was then measured at a wavelength of 635 or 605 nm.

## Figures and Tables

**Table 1:** Primer sequences for mouse tissues.

Gene	Forward	Reverse
***Ccn2***	ccacccgagttaccaatgac	gtgcagccagaaagctca
***Rab14***	gcagatttgggatacagcaggg	cagtgtttggattggtgagattcc
Gapdh *	caatgaccccttcattgacc	gacaagcttcccgttctcag

* It is common in mouse and human.

**Table 2:** Primer sequences for the human and monkey-derived cell line.

Gene	Forward	Reverse
***CCN2***	gcaggctagagaagcagagc	atgtcttcatgctggtgcag
***RAB14***	agatttgggatacggcaggac	cagtatttggattggtgagattcc
***ACAN***	gaggagagaactggagaag	gccgatagtggaatacaac
***BIP***	gtttgctgaggaagacaaaaagctc	cacttccatagagtttgctgataat
***CHOP***	gtccagctgggagctggaag	ctgactggaatctggagag
***SIAT4A***	gataagatcctgatctaccacc	ttgttctcccagtagtggtg
***18S rRNA***	gcgaattcctgccagtagcatatgctg	ggaagcttagaggagcgagcgaccaaagg

**Table 3:** Primer sequences for the amplification of the full-length and truncated forms of CCN2.

CCN2 Fragment	Forward	Reverse
***Full-length***	atccgaattccagaactgcagcgggccgtgccggtgcccg	atacggatccctcatgccatgtctccgtacatcttcctgt
***VWC-TSP1-CT***	atccgaattcgtgtgcaccgccaaagatggtgctccctgc	atacggatccctcatgccatgtctccgtacatcttcctgt
***TSP1-CT***	atccgaattcactatgattagagccaactgcctggtccaga	atacggatccctcatgccatgtctccgtacatcttcctgt
***CT***	atccgaattcaacattaagaagggcaaaaagtgcatccgt	atacggatccctcatgccatgtctccgtacatcttcctgt
***IGFBP***	atccgaattccagaactgcagcgggccgtgccggtgcccg	atacggatccgagcaccatctttggcggtgcacacgccga
***VWC***	atccgaattcgtgtgcaccgccaaagatggtgctccctgc	atacggatccagttggctctaatcatagttgggtctgggc
***TSP1***	atccgaattcactatgattagagccaactgcctggtccaga	atacggatccggatgcactttttgcccttcttaatgttct
